# Percutaneous salvage interventions in the Glenn circuit: a case series

**DOI:** 10.1186/s43044-020-00113-w

**Published:** 2020-10-31

**Authors:** Aritra Mukherji, Sanjiban Ghosh, Jayita Nandi Das, Amitabha Chattopadhyay

**Affiliations:** 1 Department of Pediatric Cardiology, Narayana Superspeciality Hospital, 120/1 Andul Road, Near Nabanna, Shibpur, Howrah, West Bengal 711103 India; 2 31A Creek Row (Nil Ratan Sarkar Sarani), 1st Floor, Kolkata, West Bengal 700014 India

**Keywords:** Bidirectional Glenn surgery, Superior vena cava syndrome, Discontinuous left pulmonary artery, Pulmonary artery stenting, Case report

## Abstract

**Background:**

In majority of children bidirectional Glenn shunt is a safe and efficacious procedure with minimal post-operative issues. Rarely, there may be dysfunction in the Glenn pathway due loss of anatomical integrity or derangements in normal physiological or hemodynamic milieu. We report 4 cases in the last 3 years (2016-2019) where complications in the Glenn circuit led to serious consequences requiring transcatheter interventions.

**Case presentation:**

Two of our patients presented with frank features of superior vena cava syndrome. One of them had right Glenn anastomotic site narrowing leading to severe obstruction along with significant left pulmonary artery origin stenosis. The other child had excessive antegrade flow impeding normal Glenn flow leading to superior vena cava syndrome. The next child in our series was initially lost to follow-up after bidirectional Glenn surgery. Later on, this child was noted to have discontinuous left pulmonary artery with perfusion only to the right lung from the Glenn. The remaining child described in this series had developed a large tortuous venous collateral post Glenn shunt leading to severe cyanosis. All the above children needed prompt percutaneous interventions to revert back to their basal state. On follow-up, the benefit was sustained in all.

**Conclusions:**

Percutaneous intervention procedures often provide a successful bailout option in various complicated situations post Glenn surgery with reasonable efficacy and safety.

## Background

Bidirectional Glenn (BDG) surgery involves end to side anastomosis of the superior vena cava (SVC) to the ipsilateral pulmonary artery [1]. It serves as the intermediate palliation in the single ventricle pathway and is also known as cavo-pulmonary shunt. The BDG circuit consists of the pulmonary arteries and the connected systemic venous system. Proper functioning of the Glenn circuit requires favorable anatomic, physiologic, and hemodynamic factors [2]. Most children remain fairly asymptomatic in the first few years after this procedure. For the majority, the only reason a child would be taken up inside a cardiac catheterization laboratory after a Glenn shunt, would be prior to Fontan completion [3]. However, in the occasional child, there may be dysfunction in BDG pathway secondary to multitude of factors and may require interim attention. In this report, we highlight few such instances where percutaneous intervention was useful to salvage the situation.

## Case presentation

### Case 1

A 9-month-old male infant presented to us with cyanosis and history of frequent spells. Detailed echocardiography revealed a diagnosis of double inlet left ventricle with normally related great arteries with severe infundibular pulmonary stenosis. The child was taken up of for BDG surgery. Intra-operative pulmonary artery (PA) pressure was 16 mm Hg and right pulsatile BDG was done. As per institutional protocol, interruption of main pulmonary artery was not done since pulmonary artery pressures were < 18 mm of Hg. Post-operative recovery was smooth and the child was discharged on oral antiplatelet agent. At 2 months follow-up, he was noted to have increased cyanosis (Spo2- 78%) with mild flow reversal on right SVC Doppler. Pulmonary vasodilator (oral sildenafil) was started but the child came back after a month with further desaturation, facial puffiness, and tachypnea. A diagnosis of SVC syndrome was made and taken up for cardiac catheterization.

**Fig. 1:**
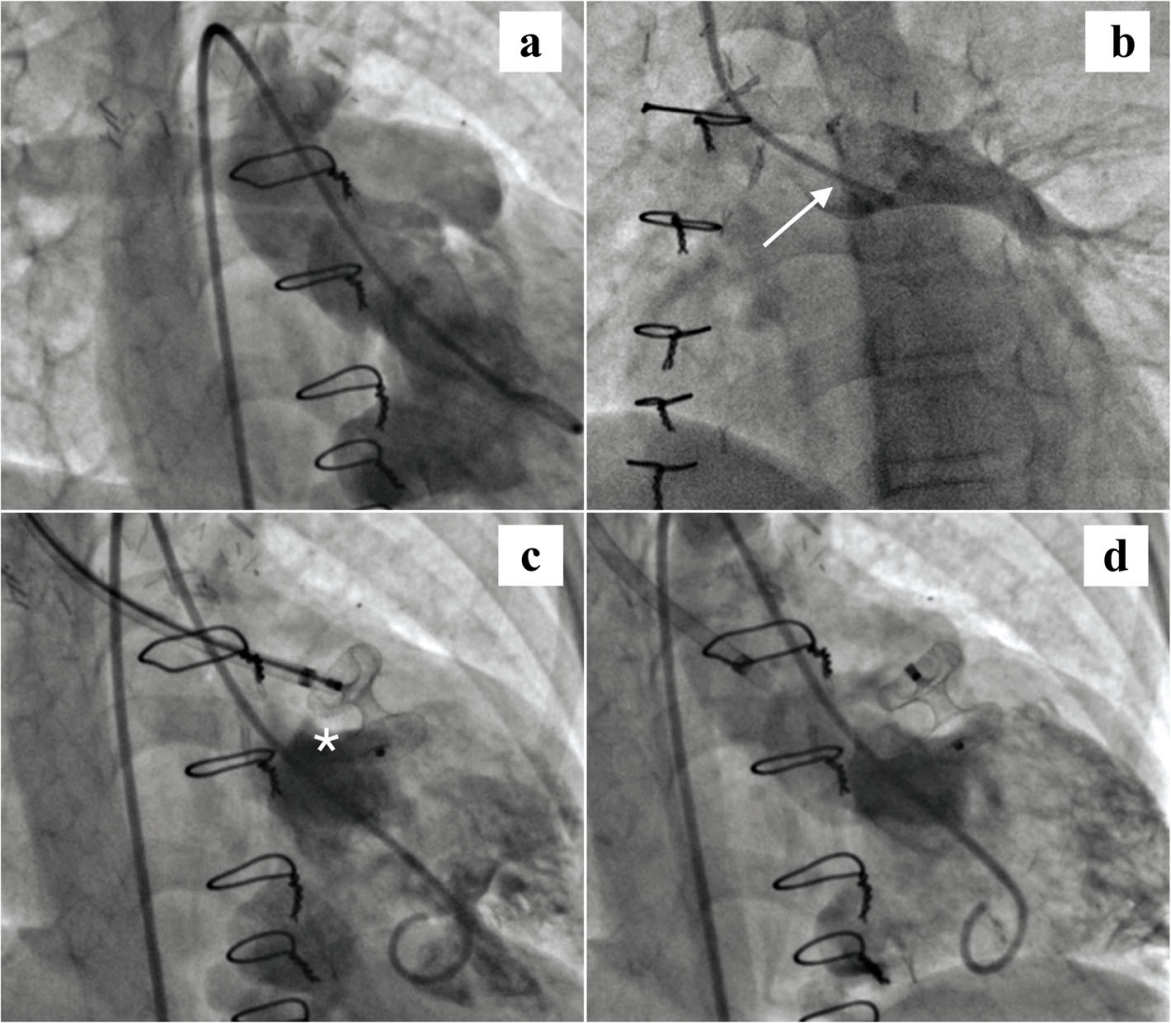
Antegrade flow occlusion: **a** Ventricular angiogram shows simultaneous and comparable opacification of both great arteries. **b** Angiogram in proximal left pulmonary artery with prominent negative clearance (white arrow), secondary to significant antegrade MPA flow. **c** 10 mm Amplatzer muscular VSD occluder with two disks deployed on either side of the muscular infundibulum (white asterix). **d** Angiogram after release shows stable device position and complete occlusion of forward flow

Right internal jugular, right femoral venous and right femoral arterial accesses were obtained. Ventricular angiogram done in shallow right anterior oblique (RAO) projection revealed similar opacification of aorta and the main pulmonary artery suggesting significant forward flow (Fig. 1a). PA was entered from jugular access and a high mean PA pressure of 21 mm Hg was observed. PA angiogram revealed no anatomical obstruction but substantial negative clearance of dye by the antegrade flow (Fig. 1b). SVC syndrome in this child was thus thought to be a consequence of antegrade flow-mediated Glenn failure. It was decided on table to close the flow percutaneously. The pulmonary valve was crossed from the jugular route using 7 Fr Amplatzer TorqVue delivery sheath (St. Jude Medical, St Paul, Minnesota, USA). A 10-mm Amplatzer muscular ventricular septal occluder (St. Jude Medical, St Paul, Minnesota, USA) was advanced through the sheath and deployed below the pulmonary valve across the narrowest segment of the muscular infundibulum (Fig. 1c). This led to an immediate fall in PA pressure to 18 mm Hg and a further fall to 16 mm Hg over the next 10 min. The occluder was released after 15 min. Ventricular angiogram post release of device showed complete occlusion of antegrade flow (Fig. 1d). In post-procedure, there was significant improvement in congestive symptoms over the course of few days. The child was continued on aspirin, and sildenafil was stopped after 3 months. He is doing well on follow-up, awaiting Fontan completion.

### Case 2

**Fig. 2:**
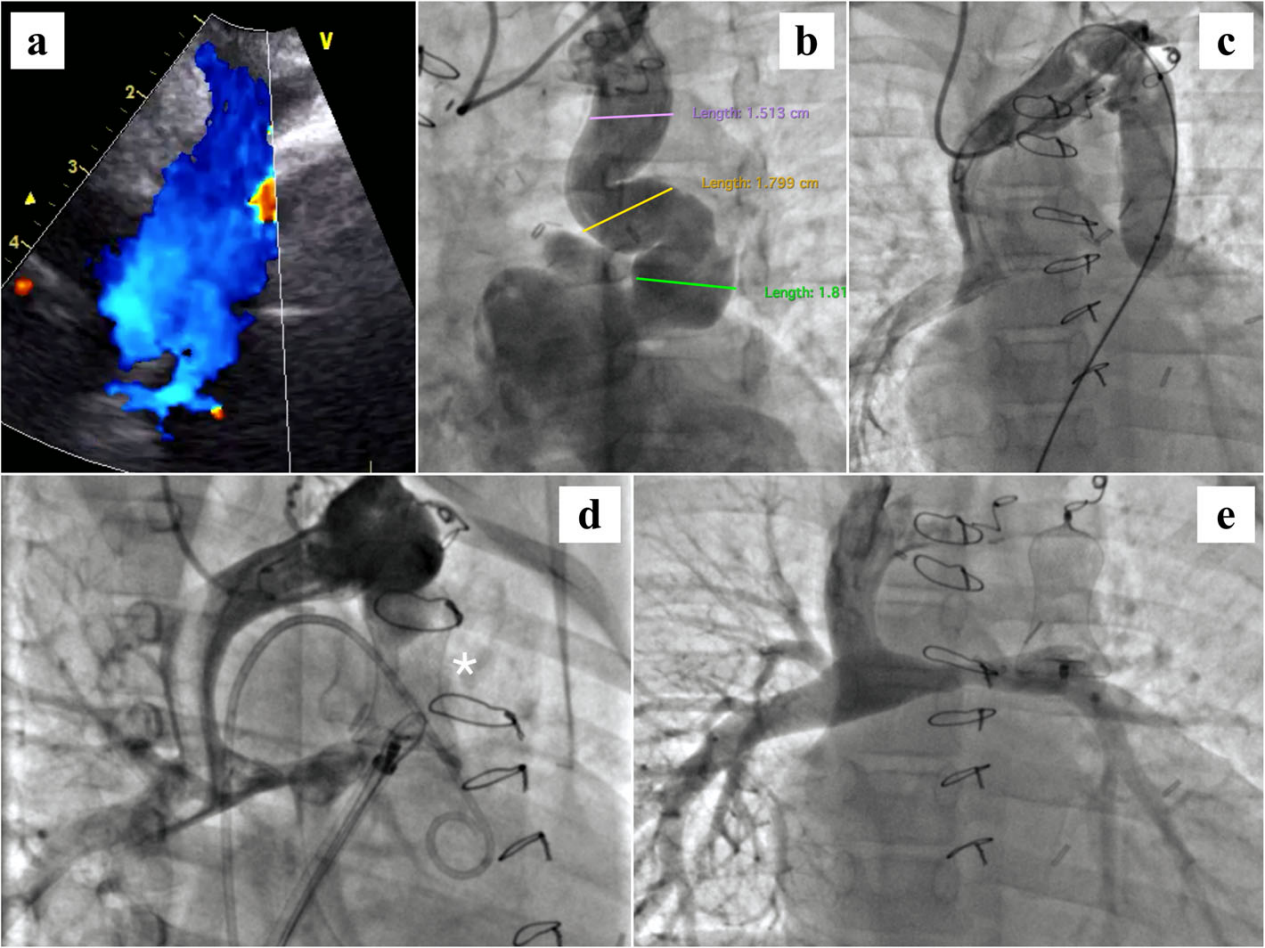
Occlusion of large venous collateral: **a** Large venous channel draining toward the heart in the region of a normal left superior vena cava. **b** Innominate vein injection shows the tortuous venous communication with multiple bends (measurements given at different segments), ultimately draining into the roof of left atrium. **c** Innominate angiogram with a 18 × 40 mm Tyshak II balloon completely occluding the venous channel shows normal drainage across the Glenn circuit. **d** Twenty-four millimeter Lifetech cera vascular plug (white asterix) deployed at the proximal end of the venous channel from femoral route. Innominate injection shows complete occlusion with normal Glenn flow. **e** Superior vena cava angiogram after release of the plug showing good flow into both branch pulmonary arteries

A 1 year 3 months old male child was diagnosed to have double outlet right ventricle with a remote ventricular septal defect and severe pulmonary stenosis. In addition, there was an obstructive cor-triatriatum. The child had cyanosis along with restricted physical activities for age. Auscultation revealed normal heart sounds and a grade 3/6 ejection murmur in the left parasternal area. On cardiac catheterization, mean PA pressure was on the higher side (18 mm Hg) but with normal trans-pulmonary gradient (3 mm Hg). The child underwent right BDG with cor-membrane resection along with atrial septectomy. He did very well on the initial few check-ups but then was lost to follow-up for nearly a year. On return, he was noted to be deeply cyanosed (Spo2- 65%) with exertional dyspnea. Transthoracic echocardiography showed good flow across right BDG anastomosis into both branch PAs. Interestingly, there was a large venous communication from the proximal bend of the left innominate vein with antegrade flow toward the heart (Fig. 2a). The draining chamber of this venous communication was not very clear on echocardiography. We reviewed the angiograms prior to BDG, but there was no evidence of any left SVC. The child was thus taken up for cardiac catheterization for further assessment.

Angiogram done from right jugular access with a catheter in the innominate vein revealed a large tortuous venous communication draining into the posterior margin of the roof of the left atrium. The venous channel had two distinct folds lower down resembling a collateral vessel. We measured a maximum dimension of 15 mm in its initial straight portion and dimensions of 17-18 mm at the tortuous lower end (Fig. 2b). We obtained the pressure in the venous communication, innominate vein, SVC, and in the branch pulmonary arteries. Mean pressure everywhere in the circuit was 11 mm Hg. Using a 18 × 40 mm Tyshak II balloon (NuMED Inc., Hopkinton, NY, USA) the venous channel was completely occluded for 15 min (Fig. 2c). This led to a substantial improvement in saturation (Spo2- 86%) without any significant rise in the Glenn pressures (11-12 mm Hg). It was hence decided to block this communication. A 24-mm Lifetech Cera Vascular plug (Lifetech Scientific, Shenzhen, China) was successfully deployed from the femoral venous route using a 9 Fr Mullins delivery sheath (Cook Medical, Bloomington, IN, USA). Angiogram after final positioning of device showed complete occlusion of the venous channel (Fig. 2d) with good flow into both branch PAs (Fig. 2e). The child was started on oral warfarin for the initial 6 months to keep the international normalized ratio (INR) above 1.5. Oral aspirin prophylaxis was continued indefinitely. The improvement in saturation was sustained during follow-up, and the child had successful Fontan completion after 2 years.

### Case 3

The third child in our series underwent right BDG and pericardial patch augmentation of left pulmonary artery (LPA) origin at 4.5 months of age. The baby had presented with early cyanosis, and echocardiography had confirmed a diagnosis of tricuspid and pulmonary atresia with severe LPA origin stenosis at the duct insertion site. On 2 months post-operative follow-up, transthoracic echocardiography showed normally functioning Glenn shunt with good flow into both branch PAs. The child again came back to us after a gap of 6 months and this time echocardiography failed to delineate any flow into LPA. CT pulmonary angiogram revealed fibrous continuity in LPA but no flow of dye into distal LPA. Subsequently, cardiac intervention was planned to recruit the LPA.

**Fig. 3:**
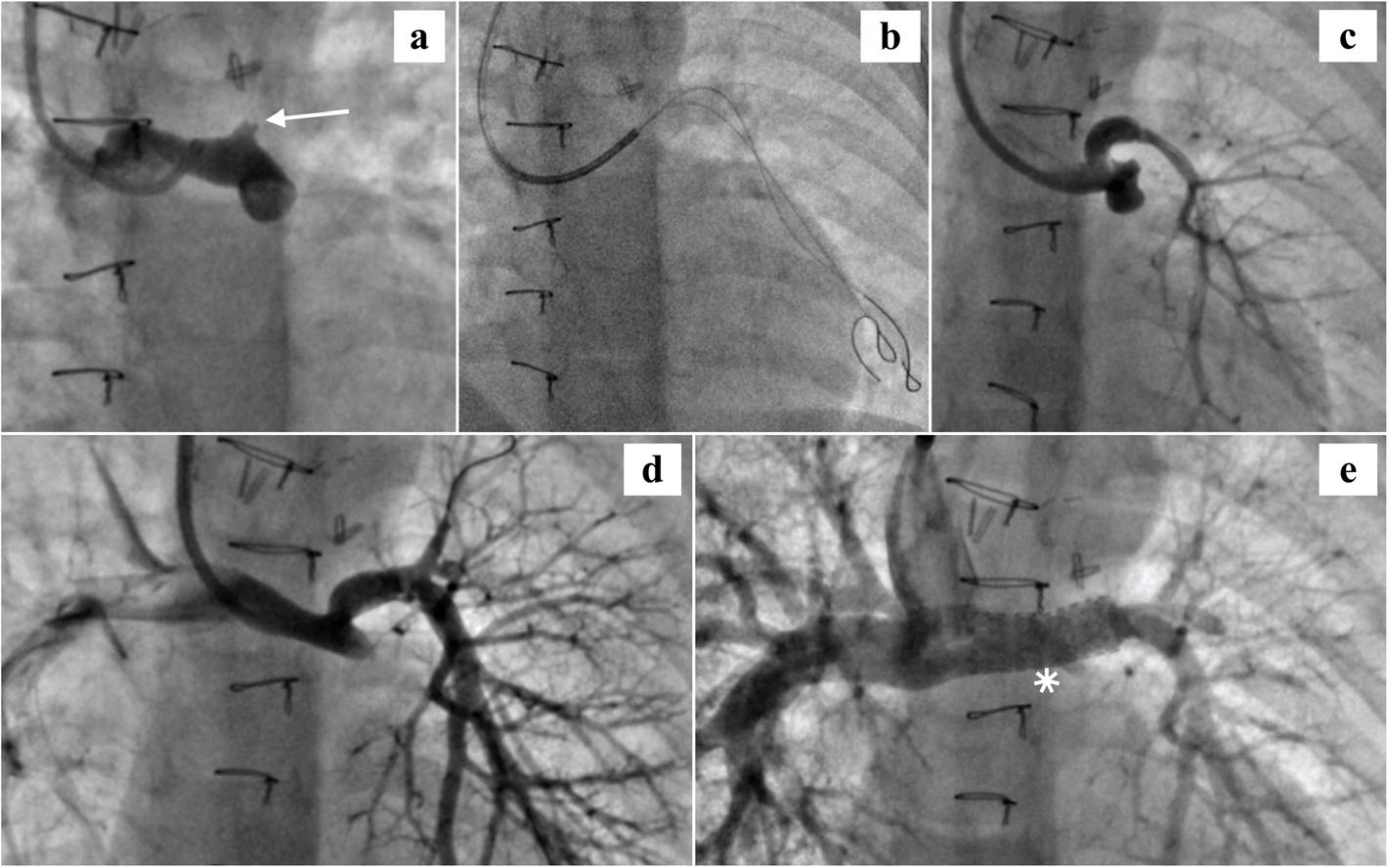
Recruitment and stenting of discontinuous left pulmonary artery: **a** Pulmonary injection revealing the blind stump of left pulmonary artery (white arrow). **b** Two coronary wires parked in distal left pulmonary artery after successful recanalization. **c** Pulmonary angiogram after graded balloon dilatation of occluded segment shows good flow into left lower lobe but poor arborization of left upper lobe. **d** Repeat injection following selective upper lobar branch balloon angioplasty. All segments of left lung well perfused. **e** Glenn injection showing the Formula stent in situ (white asterix) with good flow across it into distal left pulmonary artery

Right jugular access was obtained and initial PA angiogram showed just a blind stump of LPA, suggesting complete cut-off (Fig. 3a). We felt that the cut-off was more likely due to thrombotic occlusion rather than fibrotic stricture considering a short history of 6 months post-surgery. It was decided to attempt percutaneous recanalization of LPA. Using a ASAHI Miracle chronic total occlusion (CTO) coronary wire with a tip load of 6 g, we could successfully cross the occluded segment and the wire was parked into distal LPA. A second coronary wire was also passed into the LPA for additional support (Fig. 3b). The stenosed segment was dilated in a graded manner with 3 × 20 mm and 5 × 20 mm percutaneous transluminal coronary angioplasty (PTCA) balloons and then using a 6 × 20 mm Tyshak II balloon (NuMED Inc., Hopkinton, NY, USA). Angiogram after initial balloon dilatations revealed good opening up of proximal LPA with preservation of flow to the left lower lobe (Fig. 3c). In order to recruit the left upper lobe, selective balloon angioplasty of the upper lobar branch was done using a 4 × 20 mm PTCA balloon. Repeat angiogram revealed good flow across LPA with better peripheral arborization in all lung segments (Fig. 3d). Since the LPA had already undergone surgical-plasty, in order to ensure patency and prevent re-stenosis, we decided to stent the affected segment. A 8 × 20 mm Cook Formula 535 stent (Cook Medical, Bloomington, IN, USA) was successfully delivered from the neck approach using a 6 Fr Mullins sheath (Cook Medical, Bloomington, IN, USA). Post deployment angiogram showed good stent positioning and decent flow into both lung fields (Fig. 3e). The child was kept on Heparin infusion at 10 units/kg/h for the next 24 h and then continued on aspirin indefinitely. During 1-year follow-up, repeat cardiac catheterization revealed good flow across stent into LPA, with adequate arborization of the left lung and normal Glenn pressure. The child is awaiting Fontan completion.

### Case 4

The last child in our series had undergone right BDG at 5.5 months of age for Tricuspid atresia, normally related great vessels with severe pulmonary stenosis. Surgical LPA-plasty was needed in view of LPA origin stenosis. The child tolerated the procedure well with good initial recovery. However, she came back to us after 8 weeks with severe respiratory distress, significant cyanosis (Spo2- 55-60%), facial puffiness and frank features of SVC syndrome. Doppler interrogation of the right SVC revealed a mean gradient of 4 mm Hg across the Glenn anastomosis. Urgent cardiac catheterization was planned for further evaluation and management.

**Fig. 4.**
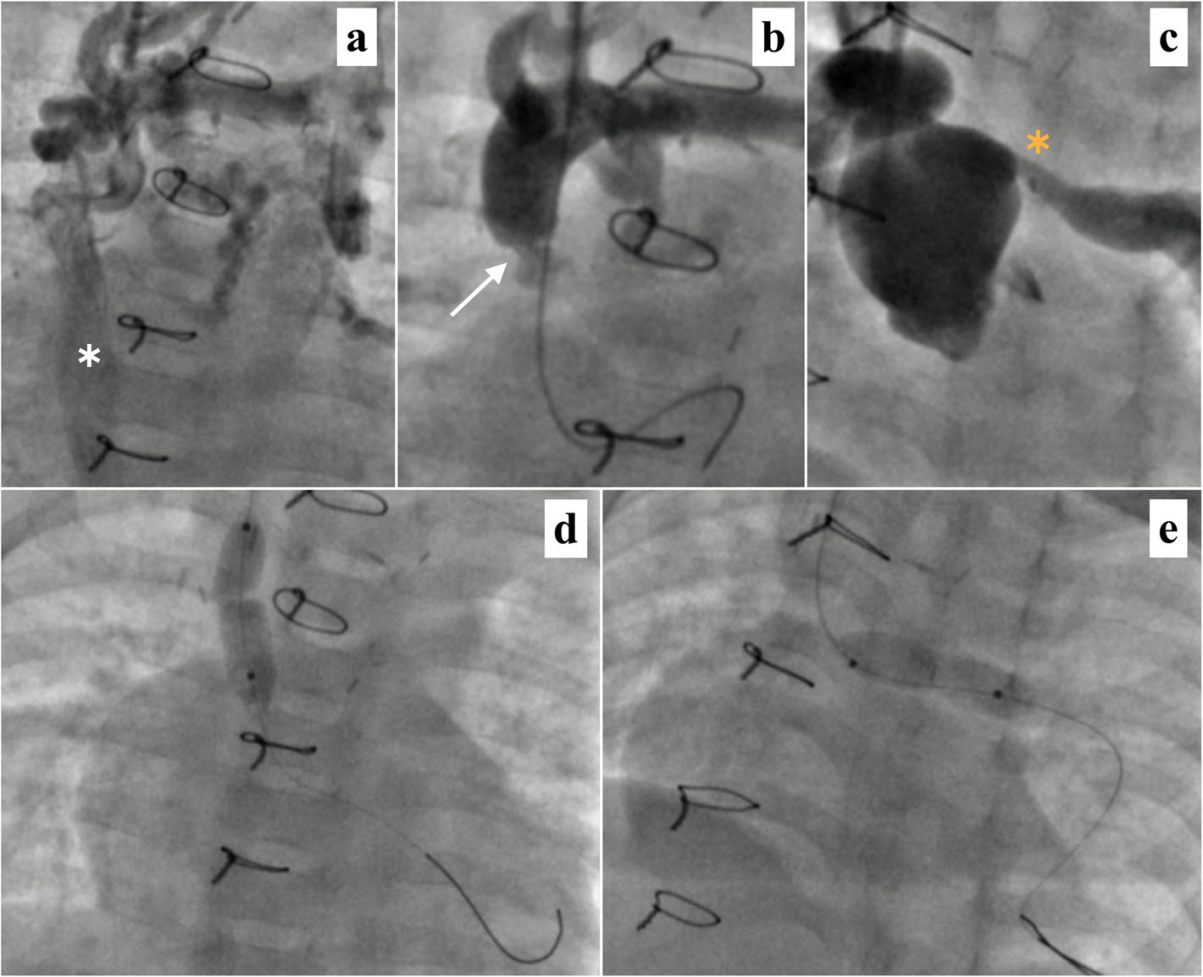
Opening-up of obstructed Glenn: **a** Injection in the upper part of right superior vena cava shows diffuse meshwork of venous collaterals mostly draining into the azygous venous system (white asterix). **b** Significant narrowing (white arrow) of the right superior vena cava and right pulmonary artery anastomotic site. **c** Pulmonary angiogram showing severe proximal left pulmonary artery stenosis (yellow asterix). **d** Balloon angioplasty of the Glenn anastomotic site. **e** Balloon angioplasty of the proximal left pulmonary artery narrowing

Right jugular venous access was obtained. Initial sheath angiogram revealed plenty of venous collaterals filling up the azygous system suggesting significant impediment to the BDG flow (Fig. 4a). The branch pulmonary arteries were not visualized well. There appeared to be a significant narrowing at the SVC-RPA anastomotic site (Fig. 4b). SVC pressure was high (22 mm Hg). We crossed the stenosed segment using a 0.035 × 260 Terumo wire and passed a multipurpose catheter into main pulmonary artery (MPA), RPA, and LPA over the said wire support. The pressure in the MPA, RPA, and distal LPA were 17 mm Hg, 17 mm Hg, and 9 mm Hg respectively, suggesting LPA stenosis. Proximal LPA stenosis was also clearly evident in the MPA angiogram (Fig. 4c). A 7 × 20 mm Tyshak II balloon (NuMED Inc., Hopkinton, NY, USA) was used to dilate the Glenn anastomotic site over a coronary wire support (Fig. 4d). The wire was then parked in distal LPA, and the LPA narrowing was successfully dilated with the same balloon (Fig. 4e). In post balloon angioplasty, there was significant fall in the Glenn pressures to 17 mm Hg. The pressure in the RPA and LPA measured 16 mm Hg and 14 mm Hg respectively. Angiogram at the end of the procedure showed persistence of venous collaterals but with good opening-up of BDG anastomotic site and proximal LPA allowing significantly improved flow into distal lung fields. The baseline saturation improved to 85% immediately after the intervention. The child’s congestive symptoms improved over the next 48 h. She is asymptomatic at present, on aspirin prophylaxis, with good BDG flow on follow-up echocardiography. The child is planned for repeat imaging after 6 months with stenting of LPA origin with a re-dilatable stent in case of restenosis.

## Discussion

BDG surgery is the first stage palliation in the univentricular pathway where SVC is anastomosed with the ipsilateral pulmonary artery in an end to side fashion. Overall, BDG is a relatively safe procedure with excellent immediate as well as delayed post-operative outcome [4, 5]. Over the years the ideal age of this procedure have come down significantly [6]. Patients undergoing BDG early in life develop progressive cyanosis with advancing age due to re-distribution of venous return, venous collateral development, and development of pulmonary arteriovenous malformations [7]. Thus, all such patients ultimately need Fontan completion beyond a certain stage. Some of the complications of BDG like pleural effusion, chylothorax, and phrenic nerve palsy occur in the immediate post-operative period. Acute Glenn obstruction is an extremely serious complication and may also cause rapid deterioration requiring immediate attention in the early post-operative period. In majority of such instances, surgical re-intervention may be necessary. Most children however fare extremely well in the initial few years after BDG. Rarely, some children may develop complications in the interim period due to various anatomic and hemodynamic factors. This is not uncommon in a developing nation like ours where late initial diagnosis, poor follow-up, and poor compliance to medications can lead to unforeseen situations, as was evident in some of our cases.

Two of our patients had features of SVC syndrome. Interestingly, the underlying pathophysiology was completely different in both of them. The last child in our cohort had obstruction in the Glenn pathway and subsequent SVC hypertension. Obstruction and decompensation may be secondary to acute Glenn thrombosis [8] or it may be due to anatomical narrowing as was seen in our child. The other patient with SVC syndrome had competitive antegrade flow hindering normal BDG flow. In our center, we follow a protocol of pulsatile BDG unless mean PA pressures are 18 mm Hg or more. We found a report by Praveen et al. where surgical MPA interruption was performed as a bailout for SVC syndrome post pulsatile BDG surgery [9]. We felt that blocking the antegrade flow with an occluder was a safe and effective approach. Various types of occluders like atrial septal occluders, duct occluders, and vascular plugs have been used for closure of antegrade flow for similar indications. Torres A et al. in their published series had 4 patients with successful occlusion of forward flow. They used duct occluder in 3 patients and atrial septal occluder in 1 patient [10]. In the Turkish series by Tunca S G et al., out of 7 children, successful antegrade flow occlusion was done using atrial septal occluders in 5 children while vascular plugs were used in 2 children [11]. For most children in the above two series, the preferred route of device delivery was from the jugular access. We used the same access to deliver a muscular ventricular septal occluder. We placed the occluder below the valve in the muscular infundibulum, far away from the Glenn circuit, thus reducing the chances of thrombus formation.

Maintaining the integrity of pulmonary arterial network is of paramount importance for single ventricle hemodynamics. Any stenosis of branch pulmonary artery can be detrimental not only for the BDG per se but also may impede future Fontan completion. One of our patients had complete LPA cut-off and without intervention would have ended up as a candidate for single lung Fontan. There are a few reports of successful stenting of branch PAs post BDG or Fontan surgery [12]. We used a Formula stent in our patient which has a low profile, good trackability, and has the potential for future re-dilatation, thus, ideal for a small child. To the best of our knowledge, this is the first report of successful transcatheter recruitment of a discontinuous branch PA in a post BDG scenario.

Veno-venous collaterals develop over time in patients with univentricular palliation [13]. These collaterals usually develop as a pop-off related to a rise in pressure within the circuit. In our second case, the child had a large tortuous collateral where the balloon occlusion test did not raise the Glenn pressures at all. Although we successfully blocked this communication, we could not initially explain this paradox. We looked back at the underlying diagnosis and this child had an obstructive cor-triatriatum. We felt that the communication might have been a persistent levo-atrio-cardinal vein [14] which was missed prior to BDG. There are various reports of successful occlusion of abnormal communications using various types of vascular plugs. Wiegand G et al. had reported successful use of vascular plugs in 9 patients with functionally univentricular heart for closure of arteriovenous and veno-venous collaterals [15]. In our case, the sizing of the plug was challenging considering the nature of the communication. We positioned the vascular plug targeting the proximal straight portion and sized it around 1.5 times the diameter of the channel and kept it as far away from the left atrium as possible to minimize the risks of embolization.

One of the challenges of percutaneous interventions in the Glenn circuit is the access and the route. Use of jugular access is well documented for interventions in congenital heart disease particularly in the setting of interrupted IVC [16]. Complex procedures like branch PA stenting or others requiring multiple hardware manipulations can become extremely cumbersome from the usual orientation with a jugular access. Positioning the child upside-down and adjusting the fluoroscopy image accordingly can often bypass this problem effectively. We have found this technique extremely useful for small children and was used with good effect in some of the cases discussed above.

## Conclusion

Complications in the Glenn pathway although rare may have serious consequences. Traditionally, most such serious complications have been tackled via surgical re-interventions. However, as evident in our series, transcatheter procedures can prove to be an excellent and safe alternative in many complicated scenarios after Glenn shunt. Such percutaneous salvage procedures assumes even more importance in these subset of patients, as they obviate the need for an additional sternotomy and cardiopulmonary pump run in children who are going to require the same again during Fontan completion.

## Data Availability

Not applicable

## Abbreviations

BDG: Bidirectional Glenn surgery
SVC: Superior vena cava
PA: Pulmonary artery
RAO: Right anterior oblique
DORV: Double outlet right ventricle
VSD: Ventricular septal defect
INR: International normalized ratio
LPA: Left pulmonary artery
CTO: Chronic total occlusion
PTCA: Percutaneous transluminal coronary angioplasty
RPA: Right pulmonary artery
IVC: Inferior vena cava
